# Knowledge about human papillomavirus and prevention of cervical cancer among women of Arkhangelsk, Northwest Russia

**DOI:** 10.1371/journal.pone.0189534

**Published:** 2017-12-13

**Authors:** Elena E. Roik, Ekaterina E. Sharashova, Evert Nieboer, Olga A. Kharkova, Vitaly A. Postoev, Jon Ø Odland

**Affiliations:** 1 Department of Community Medicine, Faculty of Health Sciences, UiT—The Arctic University of Norway, Tromsø, Norway; 2 International School of Public Health, Northern State Medical University, Arkhangelsk, Russia; 3 McMaster University, Department of Biochemistry and Biomedical Sciences, Hamilton, ON, Canada; Laboratoire National de Santé, LUXEMBOURG

## Abstract

**Background:**

Knowledge about cervical cancer (CC) risk factors and benefits of CC prevention motivates women to participate in its screening. However, several studies show that there is a significant knowledge deficit worldwide about human papillomavirus (HPV). The current study explores the level of knowledge about HPV and CC prevention in the context of sociodemographic and behavioral characteristics of women who visited an antenatal clinic in Arkhangelsk, Russia.

**Methods:**

This cross-sectional study was conducted in the city of Arkhangelsk, which seats the administrative center of Arkhangelsk County, Northwest Russia. It included women who consulted a gynecologist for any reason between January 1, 2015 and April 30, 2015, were residents of Arkhangelsk, 25 to 65 years of age and sexually active (N = 300). Student’s t-test for continuous variables and Pearson's *χ*^2^ test for categorical variables were used in the comparisons of women grouped as having either poor or sufficient knowledge. Linear regression analysis was also employed.

**Results:**

The level of knowledge about HPV and CC prevention was associated with education, parity, age of initiating of intercourse, and sources of information. After adjustment, women with university education were more likely to have higher knowledge about HPV and CC prevention compared to those with lower education.

**Conclusions:**

We observed that most participants had a sufficient level of knowledge. Educational gaps were identified that potentially could be used to tailor interventions in CC prevention.

## Introduction

Human papillomavirus (HPV) is one of the most prevalent sexually transmitted infection in both women and men worldwide and more than 100 HPV types are known [1]. They are classified as non-oncogenic (low-risk HPVs) and oncogenic (high-risk HPVs). Most such infections are transient, and more prevalent among young adults subsequent to engaging initiation in sexual activity. The most important risk factor for cervical cancer (CC) is persistent infection with high-risk HPV types [2, 3].

CC is the fourth most common cancer affecting women worldwide, with estimates of 527 624 new cases and 265 572 deaths in 2012 [4]. The highest incidence is observed in low and middle-income countries, in which CC is a major cause of cancer-related deaths. In Russia, the crude incidence of CC was 20.0 per 100 000 women in 2012 [5]. CC affects women aged 15 years and older, and its risk gradually increases with age, peaking between 45–49 years. CC is ranked as the second most common female cancer in the 15–44 year group [5]. Its incidence and mortality can be reduced by early detection of pre-invasive lesions because they respond to treatment. Cervical cytology is the most common method employed in CC screening worldwide [6]. In low and middle-income countries, many women are not screened nor followed-up regularly [7]. Low coverage, poor participation and follow-up, as well as lack of quality control measures constitute possible reasons for ineffective CC screening [8].

Knowledge about CC risk factors and benefits of its prevention motivate women to participate in screening [8]. However, several studies indicate that there is a significant HPV knowledge deficit worldwide [9–14]. Lima et al. [15] demonstrated that HPV knowledge level was associated with age, education, marital status, household income and pregnancies. Older women had higher knowledge scores compared to younger women. Moreover, women with a high school or higher education, married or with a partner, middle or high income, and previously pregnant exhibited better knowledge about HPV [15]. Hanisch et al. [8] did not find an association between HPV knowledge level and marital status, whereas age and education were associated with it.

HPV-related knowledge has been explored and described in countries worldwide [9–14], although little is known about the situation in Russia. This study aims to address this gap. We examine associations between knowledge of HPV and CC prevention and sociodemographic and behavioral characteristics of women who visited an antenatal clinic in Arkhangelsk, Russia.

## Materials and methods

### Study design, setting, participants and data collection

This cross-sectional study was conducted in the city of Arkhangelsk that seats the administrative center of Arkhangelsk County in Northwest Russia. On January 1, 2015, Arkhangelsk city had a population of 350 982 [16]. Enrolment was conducted during the period January 1, 2015 to April 30, 2015 at the *Samoylova* Clinical Maternity Hospital, which serves as an antenatal clinic for all Arkhangelsk city districts.

A sample size of 300 with an HPV prevalence of 10% was calculated to satisfy the condition (1-β) ≥ 0.80 at α = 0.05. Women who came to a gynecologist for any reason, were sexually active residents of Archangelsk and aged 25 to 65 years were invited to participate in the study. Since there are no national guidelines that regulate the cervical screening age in Russia, we adopted that of the United Kingdom NHC Cervical Screening Programme (NHSCSP). The latter recommends that routine screening be conducted during the 25–65 age interval [17]. Of the 350 women invited, 300 (86%) agreed to participate and signed the consent form. All participants completed a questionnaire (S1 Questionnaire and S2 Questionnaire). The questionnaire was designed to reflect both our study objectives and published studies, including reports by pertinent international health care agencies [18–21]. We aimed to incorporate questions that reflected current knowledge about HPV and cervical cancer prevention. Questions were formulated in such a way as to facilitate clear answers, and for some more than one response was allowed. Care was taken to ensure that the questionnaire was not too long in order to facilitate its completion while in a gynecologist’s waiting room. Prior to use, the questions were read by randomly selected women, both educated and those not receiving education beyond the basic level, to make sure that the questions were understood by all. Accordingly, appropriate adjustments were made. The questions used in the statistical analyses are provided as Supporting Information (S1 Questionnaire and S2 Questionnaire).

The questionnaire sought information about the participants’ knowledge about HPV and CC prevention (through 14 pertinent questions), as well as specific details about their sociodemographic status (age, education, marital status, parity, smoking) and their sexual behavior characteristics (age of initiating of intercourse, history of sexually transmitted infections, contraception, history of cervical cytology by the Papanicolau (Pap) test and related abnormal findings and their management). All the questions were accompanied by several possible answers to choose from. For the questions on knowledge about HPV and CC prevention, only one answer could be keyed in as correct.

### Variables

Participants’ knowledge about HPV and CC prevention was used as both a discrete variable (number of correct answers from 0 to 14) and a binary variable. For the latter, we considered having at least 50% of the questions answered correctly (7–14 out of the 14 questions) as a sufficient level of knowledge, and less than 50% of the questions answered correctly (6 or less out of the 14 questions) as a poor level. Sources of knowledge about HPV and CC prevention included TV/media, physician, or other (including family and friends). The frequency of screening for CC was categorized as once in: six months, a year (national recommendations), and in 3–5 years. The last screening the participants received was categorized into four intervals: less than 3 years ago, more than 3 years ago, never, and do not know.

Age as a variable was considered as both continues (years) and categorical (25–44 or ≥ 45 years). Education was categorized as university level or less than university level. Women were divided into three groups based on their marital status: married, cohabiting, or single (including divorced or widowed). Parity was classified as 0, 1, or ≥ 2 deliveries. Smoking was designated as yes or no (ever and never smokers).

Age of initiating intercourse was used as a continuous (years) and a categorical variable (≤17, 18–21, and > 21 years) and the number of lifetime sexual partners was recorded as three and less or more than three. History of sexually transmitted infections was categorized into ever had or never had, and contraception into four categories (use of condoms, combined oral contraceptive pill, an intrauterine device, or none).

### Data analysis

Histogram was used to describe the distribution of HPV and CC prevention knowledge (used as a discrete variable) among the study participants. Means and standard deviations for age of the participants and the age of intercourse initiation, and proportions for other sociodemographic and sexual behavior categories were calculated for each of the poor and sufficient levels of knowledge. This statistical comparison between the two levels of knowledge groups was carried out using the independent Student’s t-test for continuous variables and Pearson's *χ*^2^ test for categorical variables.

We used linear regression analysis to estimate possible associations between the level of knowledge about HPV and CC prevention (when used as a discrete variable) and sociodemographic and sexual behavior characteristics. Crude and mutually adjusted regression coefficients were calculated with 95% confidence intervals. The p-value <0.05 was considered to be statistically significant. The analysis was carried out using SPSS version 24 (SPSS Inc., Chicago, IL).

### Ethical considerations

All study participants provided written informed consent. The research was approved by the Research Ethics Committee of Northern State Medical University of Arkhangelsk, Northwest Russia Report Number 01/02-17 obtained on 01/03/2017; and the Norwegian Regional Committee for Medical and Health Research Ethics (RECNorth), Tromsø, Norway, Registered Report Number 2014/1670.

## Results

### Characteristics of study participants

The mean age of the study participants was 35.8 (9.0) years. Out of 300 participants, 163 (54.3%) had university education, 166 (55.3%) were married and 72 (24%) were cohabiting, 123 (41.0%) had one delivery, and 116 (38.7%) had two or more deliveries; and the prevalence of smoking among the participants was 13%. Almost half of the women (45.3%) reported to ever have had sexually transmitted infection. The mean age of initiating intercourse was 18.3 (2.5) years, and 50.3% reported use of contraception. Condom use was the most common method of contraception (38.3%), with 6.7% indicating the use of combined oral contraceptive pills and 5.3% intrauterine devices.

### Knowledge about human papillomavirus and cervical cancer prevention

**Fig 1:**
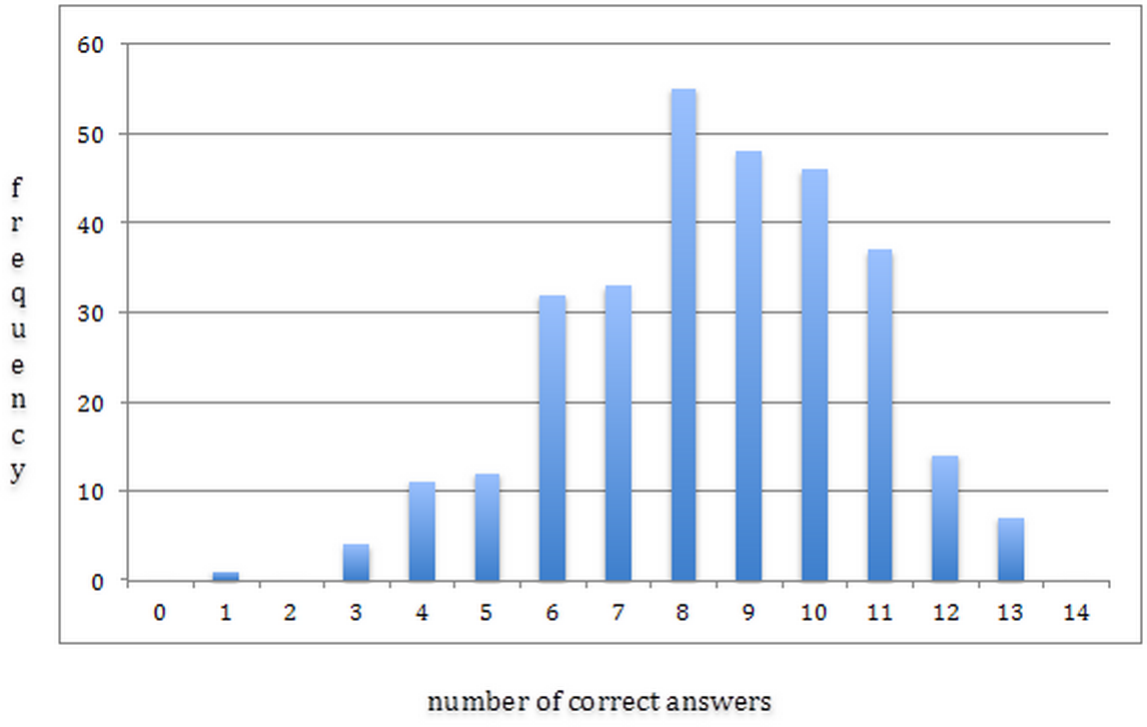
A histogram of the number of correct answers to the 14 questions about human papillomavirus (HPV) and cervical cancer prevention among women of women of Arkhangelsk (n = 300).

Fig 1 demonstrates that the number of correct answers was distributed normally among the study participants. Mean number of correct answers was 8.5 (2.2), median was 9.0, and first and third quartiles were 7.0 and 10.0 respectively.

The 14 questions used to estimate women’s knowledge about HPV and CC prevention, the answer keys, and number of women who answered correctly for each of the 14 questions are presented in Table 1.

**Table 1:** The 14 questions on knowledge about human papillomavirus (HPV), cervical cancer and its prevention; the answer keys; and the number of women who answered correctly (n = 300).

Questionnaire item	The answer keys	Number (%) of women who answered correctly
1. Have you ever heard of HPV?	yes	190 (63.3)
2. HPV is very common in women.	true	246 (81.8)
3. HPV can be passed on during vaginal sexual intercourse.	true	186 (62.0)
4. The chance of getting HPV increases with number of sexual partners.	true	224 (74.7)
5. What is the main hazard of HPV for female?	causes cervical cancer	247 (82.3)
6. Most HPV types can cleared up on their own.	true	30 (10.0)
7. A person usually does not have symptoms when infected with HPV.	true	160 (53.3)
8. Most sexually active women will never get HPV during their life.	false	202 (67.3)
9. According to Russian legislation, how often should routine screening for cervical cancer be done?	once in a year	178 (59.3)
10. Cytological smear (Pap test) can detect changes that can lead to cancer if left untreated.	true	190 (66.3)
11. HPV vaccine can prevent cervical cancer.	true	107 (35.7)
12. HPV vaccine is most effective if given to individuals who have never had sex.	true	262 (87.3)
13. Someone who has undergone HPV vaccination cannot develop cervical cancer.	false	62 (20.7)
14. Women who have undergone HPV vaccination do not need a Pap test later in their life.	false	29 (9.7)

Of the 300 responders, 74.7% understood the role of having multiple sexual partners as a risk factor of CC, and 67% were generally aware of the prevalence of HPV and sexually active people will likely contract HPV in their lifetime. However, only 35.7% of the women knew about the existence of a vaccine against HPV, and only 9.7% understood that one ought to have follow-up screening after vaccination. Moreover, 79.3% of the study participants answered incorrectly that having had HPV vaccine prevents the development of CC, and most (90%) answered incorrectly that most HPV types clear up on their own.

Out of all participants, 37.1% had undertaken screening (cytological smear from cervix, or Pap test) less than three years ago; 7.0% more than three years ago; 38.0% never had a Pap test; and 17.7% did not know. Among those who knew that screening can detect CC in its early stages, 48.5% had this test less than three years ago; 8.6% more than 3 years ago; 29.3% never had a Pap test; and 13.6% did not know (p < 0.001).

About one third of the study participants reported that their doctor was the main source of information about HPV and CC prevention. Others mentioned TV/media (53.3%) and other sources of information (12.7%).

### Associations between the level of knowledge about human papillomavirus, cervical cancer prevention and other personal characteristics

Of the 300 participants, 20.0% had a poor level of knowledge about HPV and CC prevention and 80.0% had sufficient knowledge. Selected characteristics of the study participants stratified by their level of knowledge about HPV and CC prevention are provided in Table 2.

**Table 2:** Sociodemographic and sexual behavior characteristics according to the two levels of knowledge about human papillomavirus (HPV) and cervical cancer prevention in women of Arkhangelsk (n = 300).

Variable	Level of knowledge^1^^,^^2^		P value^3^
	Poor (n = 60)	Sufficient (n = 240)	
*Age*, *years*	34.7 (7.8)	36.1 (9.2)	0.288
*Age*			0.335
25–44 years	53 (88.3)	199 (83.3)	
≥45 years	7 (11.7)	40 (16.7)	
*Education*			0.049
University	26 (43.3)	138 (57.5)	
Other	34 (56.7)	102 (42.5)	
*Marital status*			0.245
Married	29 (48.3)	137 (57.1)	
Cohabiting	17 (28.3)	45 (18.8)	
Single	14 (23.4)	58 (24.2)	
*Parity*			0.049
0 deliveries	14 (23.3)	47 (19.6)	
1 delivery	31 (51.7)	92 (38.3)	
≥ 2 deliveries	15 (25.0)	101 (42.1)	
*Smoking*			
Yes	7 (11.7)	32 (13.3)	0.731
No	53 (88.3)	208 (86.7)	
*Age of initiating of intercourse*, *years*	17.7 (1.7)	18.4 (2.7)	0.014
*Age of initiating of intercourse*			0.145
<17 years	28 (46.7)	89 (37.1)	
18–21 years	30 (50.0)	126 (52.5)	
≥22 years	2 (3.3)	25 (10.4)	
*Number of partners*			0.224
1–3	28 (46.7)	133 (55.4)	
>3	32 (53.3)	107 (44.6)	
*Sexually transmitted infections*			0.985
Never	33 (55.0)	131 (54.6)	
Ever	27 (45.0)	109 (45.4)	
*Contraception*			0.547
Condoms	25 (41.7)	90 (37.5)	
Combined oral contraceptive pill	3 (5.0)	17 (7.1)	
Intrauterine device	5 (8.3)	11 (4.6)	
No contraception	27 (45.0)	122 (50.8)	
*Source of information*			0.006
TV/media	33 (55.0)	127 (52.9)	0.773
Doctor	13 (21.7)	89 (37.1)	0.024
Social surrounding (family, friends)	14 (23.3)	24 (10.0)	0.005

^1^Number (%) for categorical variables and means (standard deviations) for continuous variables are presented according to the two levels of knowledge about HPV and cervical cancer prevention.

^2^Sufficient level of knowledge about HPV and CC prevention was defined as having at least 50% of the questions answered correctly (≥ 7 of the 14 questions). Poor level of knowledge was defined as having less than 50% of the questions answered correctly (< 7 of the 14 questions).

^3^Independent Student’s t-test for continuous and Chi-square test for categorical variables.

Women in both groups were of comparable age (in their mid-thirties). The level of knowledge about HPV and CC prevention was associated with education (p = 0.049), parity (p = 0.049), age of initiating of intercourse (p = 0.014), and source of information about HPV and CC prevention (p = 0.006) (Table 2). More specifically, women with a university education, those who had an early sexual debut, had two or more children, and/or whose physician was the primary source of information had higher levels of knowledge about HPV and CC prevention. The primary source of information about CC and its prevention was the mass media (more than 50%). Interestingly, women with adequate understanding had their physician as a source of information more frequently than those with a poor knowledge level. Moreover, the latter group received the information from their social surroundings more often compared to those with sufficient level (p = 0.005). Conversely, women with lower educational levels, nulliparous women, late sexual debut, and other sources of information on HPV and CC prevention were less informed. Associations between the level of HPV and CC prevention knowledge and age, marital status, smoking, history of sexually transmitted infections and contraception use were not evident.

Table 3 summarizes results of crude and adjusted linear regression with the knowledge about HPV and CC prevention score as the dependent variable and other characteristics as predictors.

**Table 3:** Associations between the number of correct answers to the 14 questions about human papillomavirus (HPV) and cervical cancer prevention and sociodemographic and sexual behavior characteristics of the study participants (n = 300).

Variables	Univariable^1^		Multivariable^1^^,^^2^	
	B	p-value	B	p-value
*Age*				
≥45 years	0.57	0.108	0.40	0.329
25–44 years	reference		reference	
*Education*				
University	0.56	0.029	0.65	0.021
Other	reference		reference	
*Marital status*				
Cohabiting	-0.70	0.038	-0.32	0.379
Single	-0.30	0.341	0.04	0.903
Married	reference		reference	
*Parity*				
1 delivery	0.17	0.623	0.21	0.628
≥2 deliveries	0.88	0.012	0.79	0.071
0 deliveries	reference		reference	
*Smoking*				
Yes	0.17	0.649	-0.18	0.665
No	reference		reference	
*Age of initiating of intercourse*				
≥22 years	0.89	0.062	0.62	0.214
18–21 years	0.24	0.385	0.11	0.700
<17 years	reference		reference	
*History of sexually transmitted infections*				
Ever had	0.11	0.662	0.27	0303
Never had	reference		reference	

^1^Linear regression analysis was conducted with number of correct answers to the 14 questions on knowledge about HPV and screening of cervical cancer as the dependent variable and other variables in the table as independent variables (the regression coefficients B and p-values are indicated).

^2^Regression coefficients were mutually adjusted for all variables in the table. R^2^ = 0.063, F = 1.939, df = 10, p = 0.040

Crude differences in the number of correct answers on the 14 questions about HPV and CC prevention was significant between the educational levels, and were even more pronounced after adjustment. Women with university education were more likely to have higher knowledge about HPV and CC prevention compared to women with lower educational level. Having two or more deliveries was associated with having more questions on HPV and CC prevention answered correctly when compared to nulliparous women. However, this difference was not statistically significant after adjustment. In the crude and adjusted linear regression models age, marital status, smoking, age of initiating of intercourse, number of partners and history of sexually transmitted infections were not associated with the number of correct answers to the 14 questions about HPV and CC prevention.

## Discussion

Our study demonstrates that majority of women in Arkhangelsk had sufficient level of knowledge about HPV and CC prevention, and this was associated with level of education, parity, age of initiating of intercourse, and source of information about HPV and CC prevention. After adjustment, women with university level of education were more likely to have a higher score of correct answers on knowledge about HPV and CC prevention compared to less educated women.

### Interpretation of the results and comparison with other studies

The level of knowledge and awareness about HPV and CC prevention has been suggested to be important in the development of positive approaches toward CC prevention [8]. Our results show that most of the women in our sample knew the potential consequences of having an HPV infection. Several studies report opposite results and demonstrate a significant deficit in HPV knowledge among women worldwide [8, 9, 11, 12, 15].

While many participants in our study were aware of the sexual transmission of HPV, gaps in knowledge about symptoms and treatment of HPV infection were nevertheless evident. Although HPV is one of the most common sexually transmitted infections, it is transient and thus women tend not to seek treatment. Our findings demonstrate that close to 90% of women think that HPV should be treated. This result can be partly explained by the misinformation provided by some health care professionals and pharmaceutical companies that detection of HPV requires antiviral treatment. The HPV test is commercially and widely available in Russia. Another possible explanation is a wide use of colposcopy in Russia, while the number of educational courses and amount of literature on how to perform this procedure correctly is limited [22, 23]. This can lead to an over-diagnosis of cervical lesions. Lack of guidelines and training among doctors are mentioned as pertinent factors of over diagnosis and over treatment of cervix [23].

Women in our study have exhibited good knowledge about CC risk factors. Our study also shows that knowledge about the latter and CC screening is of great importance. Thus those women who were aware that the Pap test is used for CC screening took this test more often than women who did not. However, our analysis also reveals that there was insufficient knowledge about HPV vaccination (in terms of the development of CC and need of Pap test). Although the HPV vaccine is the most effective way to prevent HPV infection, it is not widely available in Russia as it is not yet included in the Russian official vaccination program. These findings highlight a great importance of educational efforts in CC prevention.

We found no association between the level of knowledge about HPV and CC prevention and age. An earlier study reported that younger women had a higher level of knowledge of HPV and CC prevention [24]. Younger populations [24] specify that Internet and other mass media constitute their main source of pertinent information. Educating older women may facilitate their participation in CC screening, and this would help to reveal the presence of CC at earlier stages and thereby reduce the CC burden. Indeed, Williams et al. [25] report that most of the respondents with higher HPV knowledge received this information in school. This suggests that HPV education might best occur at early ages, such as in school settings or even earlier.

With respect to age, all women in our study were at risk of getting HPV infection and thus developing CC. Clearly, they constitute an age group to whom preventive measures such as CC screening must be directed. However, Tiro et al. [26] showed that older and less educated women would benefit from improved awareness of HPV and CC prevention. Our findings reinforce this, as university educational level was independently associated with a higher level of knowledge about HPV and CC prevention [8, 24]. Smoking which is a known risk factor for CC was not associated with the level of knowledge in our study [26, 27]. The relatively low smoking rate (12–13%) among our study subjects is likely the reason. A recent study in the Murmansk region of North-west Russia for the period 2006–2011 reports that the prevalence of smoking among delivering women was 25.2% prior to pregnancy [28]. Differences in age and education level might have contributed to this two-fold difference.

Risky behavior ─ specifically early age of intercourse initiation and high number of sexual partners ─ is generally accepted as increasing the risk of HPV infection [29]. We found no association between number of sexual partners and level of their knowledge about HPV and CC prevention, although individuals in our knowledge sufficient group were slightly older (p = 0.014). Interestingly, an earlier USA study indicated that neither age of intercourse initiation nor number of lifetime sexual partners were associated with knowledge scores [27].

We observed no association between the level of HPV and CC knowledge and history of sexually transmitted infections. Even though women with such history had to seek a physician about their infection, they had not received information about HPV or had forgotten it. This suggests that clinicians need to pay more attention to women`s education about HPV and HPV associated cancers, as well as other sexually transmitted infections. Neither was there an association with the type of contraception. Moreover, every second woman did not use contraception at all. One of possible explanations could be that they are more concerned about being infected with chlamydia or gonorrhea than with HPV. In doing so, they may have underestimated the seriousness of an HPV infection and as a consequence fail to seek help.

Interestingly the level of HPV and CC prevention knowledge was associated with source of information. Sufficient level of knowledge was more common among those who reported having a doctor as the primary source of information. Despite the fact that more than half of the study participants received knowledge about CC and its prevention from media and TV, there was no difference in the knowledge level in this context. Since there was a difference for physicians as the information source, we conclude that they could provide more precise and accurate knowledge about CC and its prevention. Consequently for the proper implementation and acceptability of cervical cancer screening, physicians need to provide more education on the importance of routine screening for cervical cancer prevention. In addition, educational campaigns for women are needed, since social surrounding also constitutes a source of information that leads to a poor level of knowledge.

Holcomb et al. [27] also observed this, although sexual behavior of those with higher level of knowledge did not differ from others in their study. Interestingly, Tiro et al. [26] state that women who did not trust any source of information about health were less likely to report HPV awareness.

Several Russian studies and those conducted in former Soviet Union countries have evaluated level of knowledge about HPV and CC among specific groups, such as medical students and health professionals [11, 12, 13]. All these investigations demonstrate a relatively low level of knowledge about HPV among the participants. Kahn et al. [30] describes a similar situation among USA pediatricians. These various findings indicate that educational efforts in relation to HPV infection have not been widespread. Even though scientific knowledge about HPV is on the increase (especially in terms of virus detection), a need for an increase in knowledge about HPV and CC prevention prevails in both the general population and health professionals.

### Limitations and strengths

Our study has several potential limitations. In spite of a relatively high response rate (86%), bias due to non-participation remains a possibility. Taking into account that this is a cross-sectional study and all the information was self-reported, recall bias may have occurred. Moreover, the data were collected at an antenatal health care center, and therefore a built-in selection bias may have occurred. It is possible, for example, that individuals who participated in the survey were more health conscious and curious about HPV than women in the general population. It is also important to mention that our study sample might have over represented frequent users of the health care system, and thus under represented those who live in remote and poorer regions of Arkhangelsk with less access to health care services. If true, the knowledge about HPV and CC prevention would probably be lower than what we assessed. Because of the cross-sectional nature of the study, the associations observed between the level of knowledge about HPV and CC prevention and selected behavioral characteristics cannot be interpreted as causal.

To our knowledge, this is the first study in Russia aiming to assess knowledge about HPV and CC prevention and associations between level of this knowledge and a range of sociodemographic and sexual behavioral characteristics among women. Our study population was comprised of women in the age group at risk of HPV infection, and therefore constituted the target group for CC screening and prevention.

## Conclusion

Our study provides detailed information on knowledge about HPV and CC screening among women of Arkhangelsk, Northwest Russia. We observed that most participants had a sufficient level of knowledge. Women with a university level of education displayed the highest knowledge scores about HPV and CC prevention. Health care professionals as the primary source of information are well positioned to improve women’s knowledge about HPV and CC screening, including promotion of preventive measures. However, a large proportion of participants did not know that most HPV types clear up on their own, that HPV vaccination does not avoid the development of CC nor replaces the need for a Pap test later in life. Consequently, there is need to improve knowledge about the role of CC screening and HPV vaccination in CC prevention among women of Arkhangelsk. These educational gaps might be used to tailor interventions in CC prevention. Consequently, ongoing educational campaigns that emphasize the role of HPV in CC development and importance of regular screening and HPV vaccination are recommended.

## Supporting information

S1 Questionnaire Questionnaire assessing knowledge of HPV and CC prevention and sociodemographic and behavioral characteristics of women. In Russian.
(DOC)

S2 Questionnaire Questionnaire assessing knowledge of HPV and CC prevention and sociodemographic and behavioral characteristics of women. In English.
(DOCX)

## References

[1] Center for disease control and prevention. Manual for the surveillance of vaccine-preventable disease. Chapter 5: Human papillomavirus (HPV) Available online: http://www.cdc.gov/vaccines/pubs/surv-manual/chpt05-hpv.html

[2] Ho GY, Burk RD, Klein S, Kadish AS, Chang CJ et al Persistent genital human papillomavirus infection as a risk factor for persistent cervical dysplasia. J Natl Cancer Inst 1995;87:1365–71. 7658497 10.1093/jnci/87.18.1365

[3] Schiffman M, Kjaer SK. Natural history of anogenital human papillomavirus infection and neoplasia. J Natl Cancer Inst Monogr 2003;31:14–9. 10.1093/oxfordjournals.jncimonographs.a003476 12807940

[4] Bruni L, Barrionuevo-Rosas L, Albero G, Aldea M, Serrano B, Valencia S et al. ICO Information Centre on HPV and Cancer (HPV Information Centre). Human Papillomavirus and Related Diseases in the World. Summary Report 2015–04–08 [updated 2015 December 15]. Available from: http://www.hpvcentre.net/statistics/reports/XWX.pdf

[5] Bruni L, Barrionuevo-Rosas L, Albero G, Aldea M, Serrano B, Valencia S et al. ICO Information Centre on HPV and Cancer (HPV Information Centre). Human Papillomavirus and Related Diseases in Russian Federation. Summary Report 2016–02–26 [updated 2016 June 15]. Available from: http://www.hpvcentre.net/statistics/reports/XWX.pdf

[6] Kobayashi D, Takahashi O, Hikosaka C, Okubo T, Fukui T Optimal cervical cytology mass screening interval for cervical cancer. Arch Gynecol Obstet. 2013 3;287(3):549–54. Epub 2012 Oct 11. doi: 10.1007/s00404-012-2588-8 23053319 10.1007/s00404-012-2588-8

[7] Aniebue PN, Aniebue UU Awareness and practice of cervical cancer screening among female undergraduate students in a Nigerian university. J Cancer Educ. 2010 3;25(1):106–8. doi: 10.1007/s13187-009-0023-z 20082175 10.1007/s13187-009-0023-z

[8] Hanisch R, Gustat J, Hagensee ME, Baena A, Salazar JE, Castro MV et al Knowledge of Pap screening and human papillomavirus among women attending clinics in Medellín, Colombia. Int J Gynecol Cancer. 2008 Sep-Oct;18(5):1020–6. Epub 2007 Nov 16. doi: 10.1111/j.1525-1438.2007.01131.x 18021221 10.1111/j.1525-1438.2007.01131.x

[9] Moreira ED, Oliveira BG, Ferraz FM, Costa S, Costa Filho JO, Karic G. Knowledge and attitudes about human papillomavirus, Pap smears, and cervical cancer among young women in Brazil: implications for health education and prevention. Int J Gynecol Cancer. 2006 Mar-Apr;16(2):599–603. doi: 10.1111/j.1525-1438.2006.00377.x 16681732 10.1111/j.1525-1438.2006.00377.x

[10] Massad LS, Evans CT, Wilson TE, Goderre JL, Hessol NA, Henry D et al Knowledge of cervical cancer prevention and human papillomavirus among women with HIV. Gynecol Oncol. 2010 4, 117(1): 70–76 doi: 10.1016/j.ygyno.2009.12.030 20106513 10.1016/j.ygyno.2009.12.030 PMC3100195

[11] Volchenko A.N. Dynamics of cervical cancer incidence rate and levels of awareness about its prevention among different population groups Human health and ecology. 2014 February (2): 25–30.

[12] Voloshin M.V. Level of knowledge about cervical cancer and it’s prevention among senior medical students of National medical university od Belarus. Scientific aspirations 2013 5 (5): 120.

[13] Borovikova O.I., Kucenko I.I. Borovikov I.O., Nikogda Y. V. Awareness of cervical cancer in Krasnodar. Modern problems of science and education. 2012 6: 80–82.

[14] Haesebaert J, Lutringer-Magnin D, Kalecinski J, Barone G, Jacquard AC, Regnier V, et al French women's knowledge of and attitudes towards cervical cancer prevention and the acceptability of HPV vaccination among those with 14–18 year old daughters: a quantitative-qualitative study. BMC Public Health 2012 11 27;12:1034 doi: 10.1186/1471-2458-12-1034 23186288 10.1186/1471-2458-12-1034 PMC3533507

[15] Lima EG, de Lima DB, Miranda CA, de Sena Pereira VS, de Azevedo JC, de Araulo JM, de Medeiros Fernandes TA, de Azevedo PR, Fernandes JV Knowledge about HPV and Screening of Cervical Cancer among Women from the Metropolitan Region of Natal, Brazil. ISRN Obstet Gynecol. 2013 3 31; 2013:930479 Print 2013. doi: 10.1155/2013/930479 23606981 10.1155/2013/930479 PMC3628759

[16] Territorial office of the federal state statistics service of the Arkhangelsk region. Available from: http://arhangelskstat.gks.ru/

[17] The United Kingdom NHC cervical screening program https://www.bsccp.org.uk/assets/file/uploads/resources/NHSCSP_20_Colposcopy_and_Programme_Management_(3rd_Edition)_(2).pdf

[18] Marlow LA, Zimet GD, McCaffery KJ, Ostini R, Waller J. Knowledge of human papillomavirus (HPV) and HPV vaccination: an international comparison. Vaccine. 2013; 31(5): 763–9. doi: 10.1016/j.vaccine.2012.11.083 . 23246310 10.1016/j.vaccine.2012.11.083

[19] Waller J, Ostini R, Marlow LA, McCaffery K, Zimet G. Validation of a measure of knowledge about human papillomavirus (HPV) using item response theory and classical test theory. Prev Med. 2013; 56(1):35–40. doi: 10.1016/j.ypmed.2012.10.028 . 23142106 10.1016/j.ypmed.2012.10.028

[20] World Health Organization. Human papillomavirus (HPV) and cervical cancer. Fact sheet. Available online: http://www.who.int/mediacentre/factsheets/fs380/en/

[21] Center for disease control and prevention. Human papillomavirus (HPV). Questions and answers. Available online: https://www.cdc.gov/hpv/parents/questions-answers.html

[22] Maklecova SA, Rabinkina TS Papilloma virus: opportunities for combined treatment. StatusPraesens 2015; 1(24):73–78

[23] Kataeva OA, Rogovskaya SI Cervicology, HPV,screening StatusPraesens 2014; 4(21): 19–25

[24] Capogrosso P, Ventimiglia E, Matloob R, Colicchia M, Serino A, Castagna G et al: Awareness and knowledge of human papillomavirus-related diseases are still dramatically insufficient in the era of high-coverage vaccination programs. World J Urol 2015, 33(6):873–880. doi: 10.1007/s00345-014-1379-1 25179010 10.1007/s00345-014-1379-1

[25] Williams MU, Carr MM, Goldenberg D: Public awareness of human papillomavirus as a causative factor for oropharyngeal cancer. Otolaryngol Head Neck Surg 2015, 152(6):1029–1034. doi: 10.1177/0194599815577781 25820579 10.1177/0194599815577781

[26] Tiro JA, Meissner HI, Kobrin S, Chollette V: What do women in the U.S. know about human papillomavirus and cervical cancer? Cancer Epidemiol Biomarkers Prev 2007, 16(2):288–294. doi: 10.1158/1055-9965.EPI-06-0756 17267388 10.1158/1055-9965.EPI-06-0756

[27] Holcomb B, Bailey JM, Crawford K, Ruffin MT: Adults' knowledge and behaviors related to human papillomavirus infection. J Am Board Fam Pract 2004, 17(1):26–31. 15014049 10.3122/jabfm.17.1.26

[28] Kharkova OA, Krettek A, Grjibovski AM, Nieboer E, Odland JO: Prevalence of smoking before and during pregnancy and changes in this habit during pregnancy in Northwest Russia: a Murmansk county birth registry. Reprod Health. 2016 3 8;13:18 doi: 10.1186/s12978-016-0144-x 26952100 10.1186/s12978-016-0144-x PMC4782289

[29] Kjaer SK, Dahl C, Engholm G, Bock JE, Lynge E, Jensen OM: Case-control study of risk factors for cervical neoplasia in Denmark. II. Role of sexual activity, reproductive factors, and venereal infections. Cancer Causes Control 1992, 3(4):339–348. 1617121 10.1007/BF00146887

[30] Kahn JA, Zimet GD, Bernstein DI, Riedesel JM, Lan D, Huang B et al Pediatricians' intention to administer human papillomavirus vaccine: the role of practice characteristics, knowledge, and attitudes. J Adolesc Health. 2005;37:502–510. doi: 10.1016/j.jadohealth.2005.07.014 16310128 10.1016/j.jadohealth.2005.07.014

