# Supplementary material for: Knowledge about human papillomavirus and prevention of cervical cancer among women of Arkhangelsk, Northwest Russia
Source: PLoS One. 2017 Dec 13;12(12):e0189534. doi: 10.1371/journal.pone.0189534 (PMC5728530; doi:10.1371/journal.pone.0189534)
Supplement: S1 Questionnaire — In Russian. (DOC) [file pone.0189534.s001.doc]

**Номер участника исследования: _______________________________**

**1. Полных лет ______________**

**2. Ваше образование**

- среднее (школа, гимназия)
- средне-специальное (техникум, колледж)
- высшее (университет)
- другое (укажите) _______

**3. Ваше семейное положение**

- состою в зарегистрированном браке
- не замужем
- проживаю с партнером
- в разводе / вдова
- другое (укажите) _________

**4. В каком возрасте Вы начали половую жизнь?________**

**5. Сколько сексуальных партнеров у Вас было?**

- 1-3
- Больше 3

**6. Вы были беременны (включая выкидыши и аборты)?**

- Да:
- Количество родов _______
- Количество абортов ________
- Количество выкидышей _________
- Нет

**8. Вы курите?**

- Да (укажите сколько лет ___________)
- Нет

**9.** **Пользуетесь ли Вы какими-либо методами защиты от беременности?**

- Да:
- Гормональные контрацептивные препараты
- Презерватив
- Внутриматочная спираль
- Другое _____________________
- Нет

**11. Были ли у Вас когда-нибудь инфекции передающиеся половым путем?**

- Да
- Нет
- Не помню/не знаю

**12. До заполнения этой анкеты Вы когда–нибудь слышали о вирусе папилломы человека?**

- Да
- Нет

**13.** **Если Вы ответили ДА на предыдущий вопрос, пожалуйста, отметьте, какой из источников информации Вы использовали чаще**

- ТВ, интернет, газеты или журналы, радио
- Врач
- Семья или друзья

**14. ВПЧ одна из самых часто встречаемых инфекций передающихся половым путем у женщин**

- Верно
- Неверно

**15. Основной путь передачи вируса папилломы человека – половой путь**

- Верно
- Неверно

**16. Большое количество половых партнеров в течение жизни повышает риск развития рака шейки матки**

- Верно
- Неверно

**17. Вирус папилломы человека может вызвать рак шейки матки**

- Верно
- Неверно

**18. Вирус папилломы человека может пройти без назначения лечения**

- Верно
- Неверно

**19. При заражении вирусом папилломы человека не бывает симптомов**

- Верно
- Неверно

**20. Большинство сексуально активных женщин никогда в течение жизни не встретятся с вирусом папилломы человека**

- Верно
- Неверно

**21. Cогласно Российского законодательства, как часто необходимо обследоваться на рак шейки матки**

- 1 раз в полгода
- 1 раз в год
- 1 раз в 3 года
- 1 раз в 5 лет

**22. Цитологическое исследование соскоба с шейки матки (мазок на “раковые клетки”) может определить изменения на ней, которые, если их не лечить, приведут к развитию рака шейки матки**

- Верно
- Неверно

**23. Вакцина для профилактики папилломавирусной инфекции может защитить Вас от рака шейки матки**

- Верно
- Неверно

**24. Вакцинация наиболее эффективна, если проводится до начала половой жизни**

- Верно
- Неверно

**25. У людей, прошедших вакцинацию, никогда не разовьется рак шейки матки**

- Верно
- Неверно

**26. После проведения вакцинации женщинам не нужно проходить цитологическое исследование на рак шейки матки**

- Верно
- Неверно

СПАСИБО
