# Supplementary material for: Knowledge about human papillomavirus and prevention of cervical cancer among women of Arkhangelsk, Northwest Russia
Source: PLoS One. 2017 Dec 13;12(12):e0189534. doi: 10.1371/journal.pone.0189534 (PMC5728530; doi:10.1371/journal.pone.0189534)
Supplement: S2 Questionnaire — In English. (DOCX) [file pone.0189534.s002.docx]

**Study code: ____________________________________**

**1. How old are you?** ______________Years

**2. What is your education level?**

- Secondary school
- College
- University
- Other (specify)_______

**3. What is your marital status?**

- Married
- Single
- Cohabiting
- Divorced or widowed
- Other______________ (specify)

**4. How old were you when you first had your sexual intercourse?**

______________Years

**5. How many sexual partners have you had during your lifetime?**

- 1-3
- More than 3

**6. Have you ever been pregnant? (Including abortions and miscarriages)**

- Yes
  - Number of deliveries _______
  - Number of abortions _______
  - Number of miscarriages _______
- No

**8. Do you smoke?**

- Yes (specify for how many years _________)
- No

**9. Do you use contraception?**

- Yes
  - Hormonal contraceptive pills
  - Condom
  - Intrauterine device
  - Other (specify) _____________
- No

**11. Have you ever had sexually transmitted diseases?**

- Yes
- No
- Do not remember or Do not know

**12. Before participating in this survey, have you ever heard about human papilloma virus (HPV)?**

- Yes
- No

**13. If you have answered «YES» on previous question, please specify your main source of information:**

- TV, internet, newspaper or magazine, radio
- Doctor
- Family or friends

**14. Human papilloma virus (HPV) is very common in women**

- True
- False

**15. Human papilloma virus (HPV) can be transmitted during vaginal sexual intercourse.**

- True
- False

**16. The larger the number of sexual partners, the greater is chance of getting human papilloma virus (HPV)**

- True
- False

**17. Human papilloma virus (HPV) is a known risk factor for the development of cervical cancer.**

- True
- False

**18. Most HPV types can clear up on their own if left untreated.**

- True
- False

**19. A person usually does not have symptoms when infected with HPV.**

- True
- False

**20. Most sexually active women will never get HPV during their life.**

- True
- False

**21. In accordance with the Russian legislation how often routine screening for cervical cancer should be done?**

- Once in six months
- Once in a year
- Once in three years
- Once in five years

**22. Сytological cervix smear (Pap test) can detect changes that can lead to cancer if left untreated?**

- True
- False

**23. HPV vaccine can prevent cervical cancer?**

- True
- False

**24. HPV vaccination is most effective when given prior to the first sexual intercourse.**

- True
- False

**25. Someone who has undergone HPV vaccination cannot develop cervical cancer.**

- True
- False

**26. Women who have undergone HPV vaccination do not need a Pap test later in life.**

- True
- False
